# *Pseudomonas aeruginosa* Eliminates Natural Killer Cells via Phagocytosis-Induced Apoptosis

**DOI:** 10.1371/journal.ppat.1000561

**Published:** 2009-08-28

**Authors:** Jin Woong Chung, Zheng-Hao Piao, Suk Ran Yoon, Mi Sun Kim, Mira Jeong, Suk Hyung Lee, Jeong Ki Min, Jae Wha Kim, You-Hee Cho, Jin Chul Kim, Jeong Keun Ahn, Kyoon Eon Kim, Inpyo Choi

**Affiliations:** 1 Cell Therapy Research Center, Korea Research Institute of Bioscience and Biotechnology, Yuseong, Daejeon, Republic of Korea; 2 Department of Biological Science, Dong-A University, Busan, Republic of Korea; 3 Department of Biochemistry, College of Natural Sciences, Chungnam National University, Daejeon, Republic of Korea; 4 Department of Functional Genomics, University of Science and Technology, Daejeon, Republic of Korea; 5 Antibody Therapy Research Center, Korea Research Institute of Bioscience and Biotechnology, Daejeon, Republic of Korea; 6 Genome Research Center, Korea Research Institute of Bioscience and Biotechnology, Yuseong, Daejeon, Republic of Korea; 7 Department of Life Science, College of Natural Science, Sogang University, Seoul Republic of Korea; 8 Department of Microbiology, School of Bioscience and Biotechnology, Chungnam National University, Daejeon, Republic of Korea; University of Alabama at Birmingham, United States of America

## Abstract

*Pseudomonas aeruginosa* (PA) is an opportunistic pathogen that causes the relapse of illness in immunocompromised patients, leading to prolonged hospitalization, increased medical expense, and death. In this report, we show that PA invades natural killer (NK) cells and induces phagocytosis-induced cell death (PICD) of lymphocytes. *In vivo* tumor metastasis was augmented by PA infection, with a significant reduction in NK cell number. Adoptive transfer of NK cells mitigated PA-induced metastasis. Internalization of PA into NK cells was observed by transmission electron microscopy. In addition, PA invaded NK cells via phosphoinositide 3-kinase (PI3K) activation, and the phagocytic event led to caspase 9-dependent apoptosis of NK cells. PA-mediated NK cell apoptosis was dependent on activation of mitogen-activated protein (MAP) kinase and the generation of reactive oxygen species (ROS). These data suggest that the phagocytosis of PA by NK cells is a critical event that affects the relapse of diseases in immunocompromised patients, such as those with cancer, and provides important insights into the interactions between PA and NK cells.

## Author Summary

Phagocytic leukocytes, including neutrophils and macrophages, are critical for innate immunity against invading bacteria. Binding and internalization of bacteria by these immune cells stimulates a variety of anti-microbial activities. Although the immune cells are specialized for elimination of bacteria, cellular apoptosis by bacterial phagocytosis has emerged as an important mechanism of pathogenesis. NK cells are non-phagocytic lymphocytes that are responsible for innate immunity via elimination of virus or bacteria-infected cells, as well as transformed cells. We found that PA invades NK cells and that this phagocytic event results in the generation of ROS within the NK cells, leading to apoptosis. The elimination of NK cells, at least in part, may be responsible for the relapse in PA-infected cancer patients. Based on these findings, studies on the interactions between bacterial determinants and host receptors should provide further insight into the mechanisms of bacterial pathogenesis.

## Introduction

Infectious complications are one of the major causes of morbidity and mortality in immunocompromised patients, despite recent advances in therapeutic approaches and supportive care. Among the infectious agents, the increasing incidence of PA is a worldwide problem, particularly in patients with leukemia and in hematopoietic stem cell transplantation recipients [1],[2],[3]. PA is a multi-drug resistant, Gram-negative, opportunistic pathogen and is associated with significant morbidity and mortality [4],[5]. PA constitutes the major cause of prolonged hospitalization, severe illness, death, and increased cost for immunocompromised patients. A high mortality rate occurs in patients with underlying disease, such as cystic fibrosis and cancer [6],[7].

PA pathogenesis involves the production of a variety of toxic products, including alkaline protease (AP), elastase [8], and several Type III system-dependent exotoxins that include Exo A, Exo T, and Exo U [9],[10]. AP and elastase have previously been implicated in the inhibition of NK cell activity [8], and the exotoxins have been reported to induce apoptosis of phagocytes, such as dendritic cells [11], macrophages [12], and neutrophils [13]. Apoptosis and shedding of the infected, apoptotic cells may be beneficial to the survival of the host organism [14]. However, apoptosis of lymphocytes by bacterial infection has detrimental effects on host survival [15].

NK cells are lymphocytes that mature from hematopoietic stem cells (HSC) in the bone marrow (BM) [16]. Upon activation, they can eliminate leukemic cells, as well as pathogen-infected or transformed cells, either directly or indirectly through the release of cytokines and chemokines [17],[18].

Previous studies indicate that upon infection with PA, NK cells can produce interferon-γ that may assist in clearing the bacteria [19]. However, a negative role of NK cells in the regulation of PA infection has also been reported [20]. Furthermore, NKG2D and substance P have been shown to be important in host defense against PA infection [19],[21], supporting the involvement of NK cells in resistance to such infections. However, little is known about the exact mechanisms or interactions between NK cells and PA during infection.

In this report, we show for the first time that PA invades and eliminates NK cells *in vivo* and *in vitro* by induction of apoptosis via ROS generation. The reduction in NK cell number by PA invasion led to the aggravation of metastasis in a tumor-bearing animal model. Thus, the capability of PA to induce apoptosis of NK cells may be an important factor in the relapse of illness, as well as in the initiation of infection, bacterial survival, and escape from the host immune response.

## Results

### *Pseudomonas aeruginosa* K (PAK) increases tumor metastasis by suppressing NK cells *in vivo*

**Figure 1:**
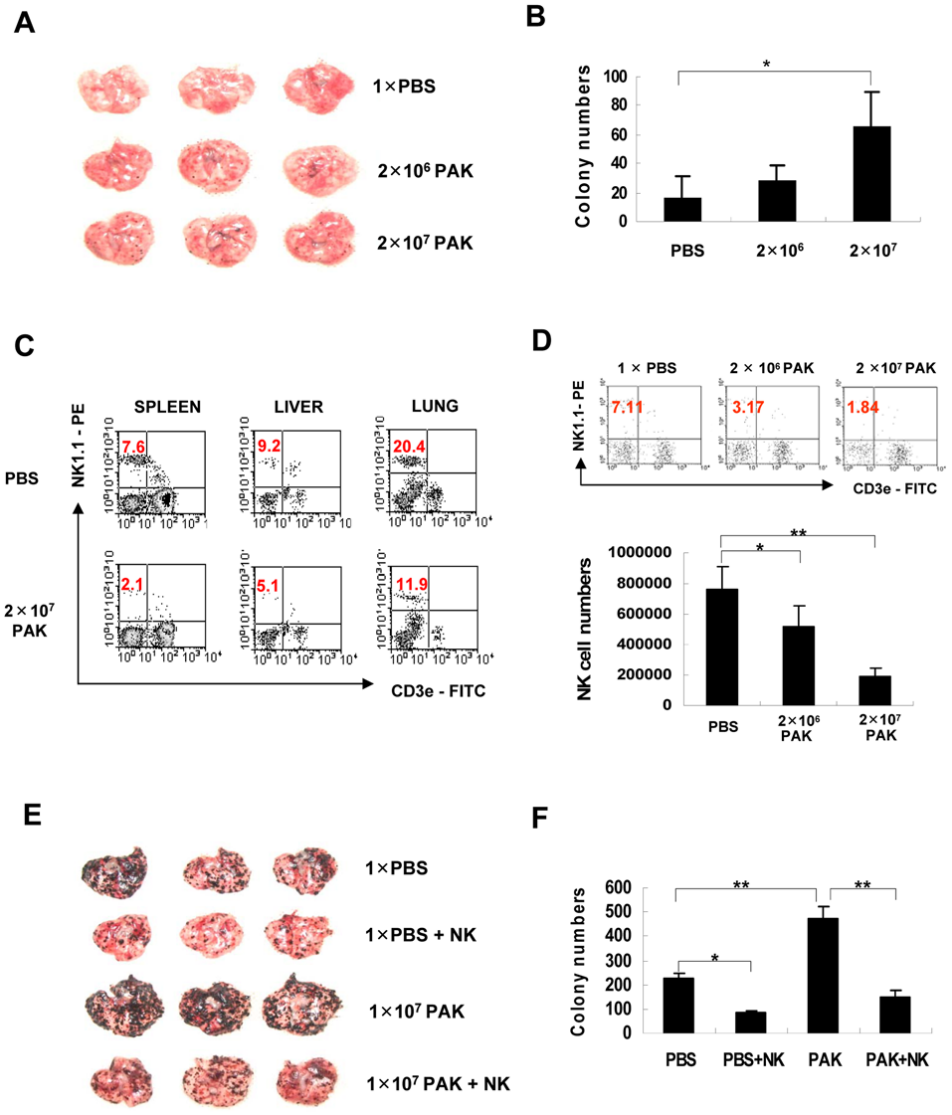
Role of NK cells in metastasis in *P. aeruginosa*-infected mice. (A&B) C57BL/6 mice were inoculated *i.v.* with 1×PBS or 1×10^7^ PAK and then with 1×10^4^ B16-F10 melanoma cells at 2 days post-infection (DPI). (A) Representative photographs of lungs from mice with metastasized colonies of B16-F10 cells 2 weeks after infection. (B) The numbers of surface metastatic foci were counted. Values represent means±SD of triplicate determinations (n = 5) (*p<0.05). (C) C57BL/6 mice were infected with 2×10^7^ PAK. Mice were sacrificed at 24 h post-infection and single cell suspensions from the spleen, liver, and lung were stained with PE-conjugated anti-NK1.1 and FITC-conjugated anti-CD3. The percentage of NK cells (NK1.1^+^/CD3^−^) in the gated lymphocyte population was determined according to their size and granularity. The data shown are representative of at least five independent experiments that yielded similar results. (D) Mice were infected with the indicated number of PAK, splenic NK cells were analyzed by flow cytometry (upper panel), and the total number of NK cells in spleens was calculated (lower panel). Values presented are means±SD of triplicate determinations (n = 3) (*p<0.05, **p<0.005). (E&F) To investigate the inhibitory effect of NK cells on PAK-induced metastasis, a larger number of melanoma cells (1×10^5^) was *i.v.* injected, and adoptive transfer of NK cells was performed via *i.v.* injection at 3 DPI. (E) Representative pictures of metastasized melanoma colonies at 2 weeks. (F) The numbers of surface metastatic foci were counted. Values presented are means±SD of triplicate determinations (n = 5) (*p<0.05, **p<0.005).

PA infection has been implicated in prolonged hospitalization and shortened lives of cancer patients [7],[22]. Thus, we analyzed the effects of PA infection on tumor metastasis in an animal model using a PAK strain. When B16-F10 melanoma cells were *i.v.-* injected into C57BL/6 mice after PAK infection, the number of metastasized tumor colonies in the lungs was significantly increased compared with the PBS-injected control (Fig. 1A, B), suggesting that metastatic tumor growth may be aggravated by PAK infection in cancer patients. We next examined the populations of various immune cells in the spleen after PAK infection. A significant reduction in the number of NK cells in the spleen, in addition to a decrease in the number of macrophages, was observed following PAK infection. However, the number of total splenocytes and other immune cells, such as T or B cells, was not significantly altered (Table 1), suggesting that NK cells might be specifically involved in PAK-induced metastasis. In fact, the reduction in the number of NK cells after PAK infection not only occurred in the spleen, but also in other tissues, such as the liver and lung (Fig. 1C). Next, we investigated whether the reduction in NK cells was specifically due to PAK infection by analyzing NK populations after *i.v.* injection of various doses of PAK. The results showed that the reduction in the number of splenic NK cells was PAK-dose dependent, with a loss of up to approximately 80% of the NK cells in 24 h at the dose of 2×10^7^ PAK, in terms of population and absolute number (Fig. 1D). These results further suggest that PAK infection significantly and specifically reduces the NK cell population, although the number of macrophages were also altered, as previously reported [23].

**Table 1:** Specific elimination of NK cells by *P. aeruginosa*.

	NK1.1^+^/CD3e^−^ (NK)	NK1.1^+^/CD3e^+^ (NKT)	NK1.1^−^/CD3e^+^ (T)	Ly6-G and Ly-6C^+^ (Gr-1)	CD11b^+^ (Mac-1)	B220^+^/CD19^+^ (B)	Splenocytes
**1×PBS**	7.64±1.43	2.39±0.55	48.31±14.04	32.17±4.38	22.34±0.84	35.95±4.32	265.30±34.69
**2×10^6^ PAK**	5.16±1.38	1.99±0.55	61.94±9.98	38.36±5.98	20.65±2.31	37.76±6.66	277.43±47.88
**2×10^7^ PAK**	1.87±0.58*	1.43±0.53	48.03±10.11	28.98±1.76	14.62±0.28*	30.53±8.11	230.76±17.09

Absolute numbers of immune cells in total splenocytes (×10^5^).

Comparative statistical analyses of immune cell population in spleen at 24 h post infection of PAK were shown in Table 1. The numbers of immune cells including NK cells (NK1.1^+^/CD3e^−^), NKT cells (NK1.1^+^/CD3e^+^), T cells (NK1.1^−^/CD3e^+^), Neutrophils (Ly6G and Ly6C^+^), Macrophage (CD11b^+^) and B cells (B220^+^/CD19^+^) were determined by calculating the absolute numbers of each cell type from the FACS profiles and total cell numbers in the tissues. Results are expressed as mean±SD of three individual experiments (n = 3, *p<0.05).

Because NK cells play a critical role in tumor clearance and defective NK cell activity results in tumor metastasis [17], we examined the role of NK cells in PAK-induced metastasis in a mouse model by performing adoptive transfer of NK cells after PAK infection. As expected, adoptive transfer rescued the diminished tumor clearance activity and restored it to control levels (Fig. 1E, F). Taken together, these findings suggest that PAK infection eliminates NK cells and subsequently results in increased tumor metastasis.

### Impairment of NK cell activity by PA infection

**Figure 2:**
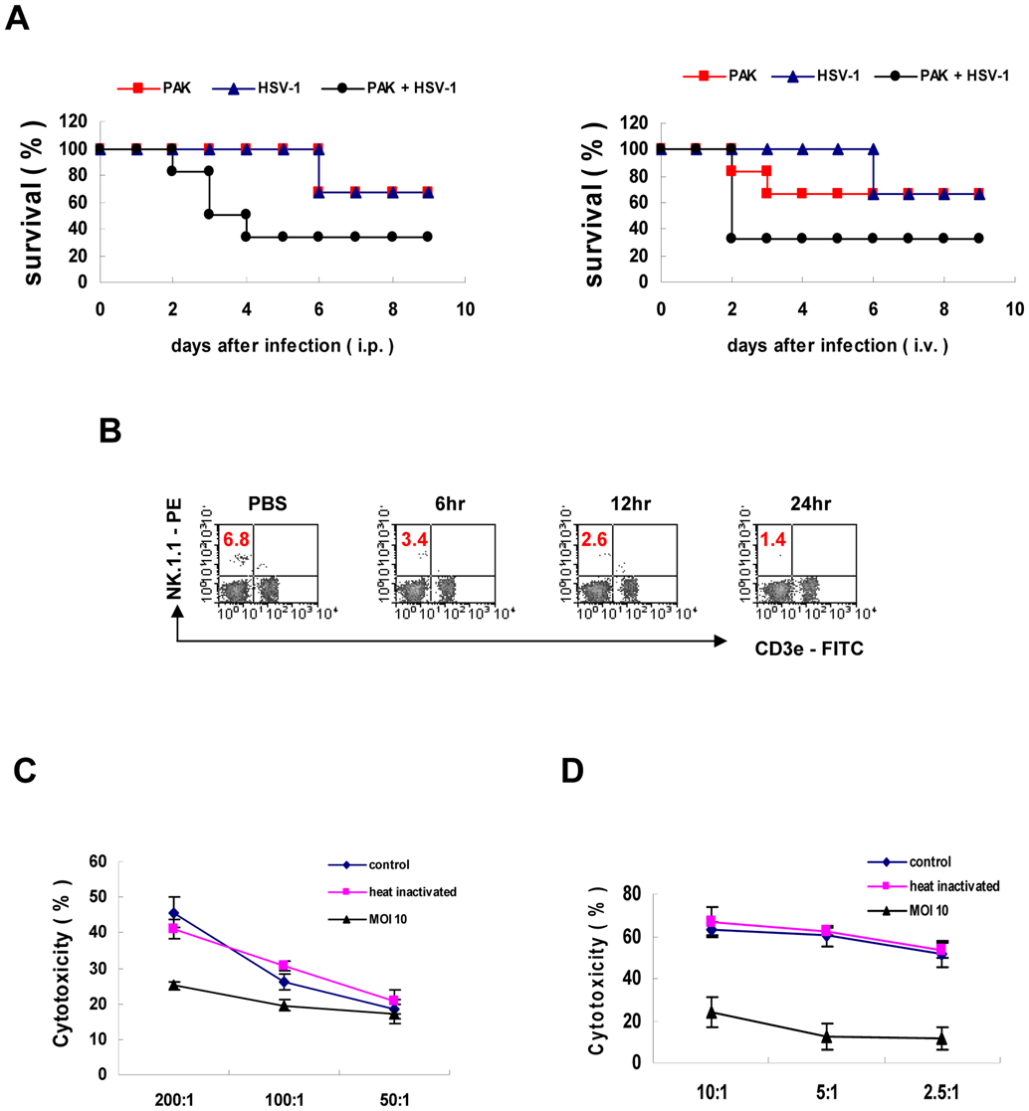
Impairment of NK cell activity by PA infection. (A) PA-induced NK cell depletion results in increased susceptibility to HSV-1. On day 0, C57BL/6 mice were *i.v.* inoculated with or without 3×10^7^ PAK. Subsequently, 1×10^7^ PFU HSV-1 or PBS was injected *i.p.* (left) or *i.v.* (right) 2 days after PAK infection (n = 6). (B) C57BL/6 mice were *i.v.-*inoculated with 2×10^7^ PAK, and splenocytes were isolated at the indicated times post-infection. The NK cell population was analyzed by flow cytometric analysis. (C & D) Freshly isolated splenocytes (C) or NK92 cells (D) were infected with live or heat-inactivated (80°C, 30 min) PAK for 12 h and then subjected to the ^51^Cr release assay using YAC-1 or K562, respectively, as target cells. Prior to the assay, the effector cells were pretreated with IL-2 (20 ng/ml) for 12 h. Values represent the means±SD of triplicate determinations (n = 5).

Next, to investigate whether a reduction of NK cells by PA infection is directly involved in NK cell function, we examined the activity of NK cells after PA infection *in vivo* and *in vitro*. Depletion of NK cells has been known to reduce resistance to herpes simplex virus 1 (HSV-1) infection [24]. Thus, we infected C57BL/6 mice with or without PAK, subsequently injected HSV-1 *i.p.* or *i.v.* 2 days later, and measured the survival rate of the mice. In both cases, Kaplan-Meier survival plots showed that the survival rates of the mice were prominently decreased when the mice were infected with PAK prior to HSV-1 infection, compared with mice infected with PAK or HSV-1 alone (Fig. 2A). The decline in resistance against HSV-1 observed in mice infected with PAK may be due to the reduction in NK cell number caused by PAK infection. Next, to investigate the effects of PAK infection on NK cytolytic activity, we performed *in vitro* ^51^Cr-release assays using PAK-infected cells. At 12 h post-infection, the population of NK cells in the spleen decreased to approximately 40% of the control (Fig. 2B). Accordingly, the cytotoxic assay revealed an approximately 40–50% reduction in the activity of PAK-infected splenocytes compared with control or heat-killed PAK-treated splenocytes (Fig. 2C). Moreover, NK92 cell lines also showed significantly reduced cytotoxicity when the cells were infected with PAK *in vitro* (Fig. 2D). Taken together, these results demonstrate that PAK infection reduces the number and functions of NK cells *in vivo* and *in vitro*.

### Direct effect of PAK on NK cell apoptosis

**Figure 3:**
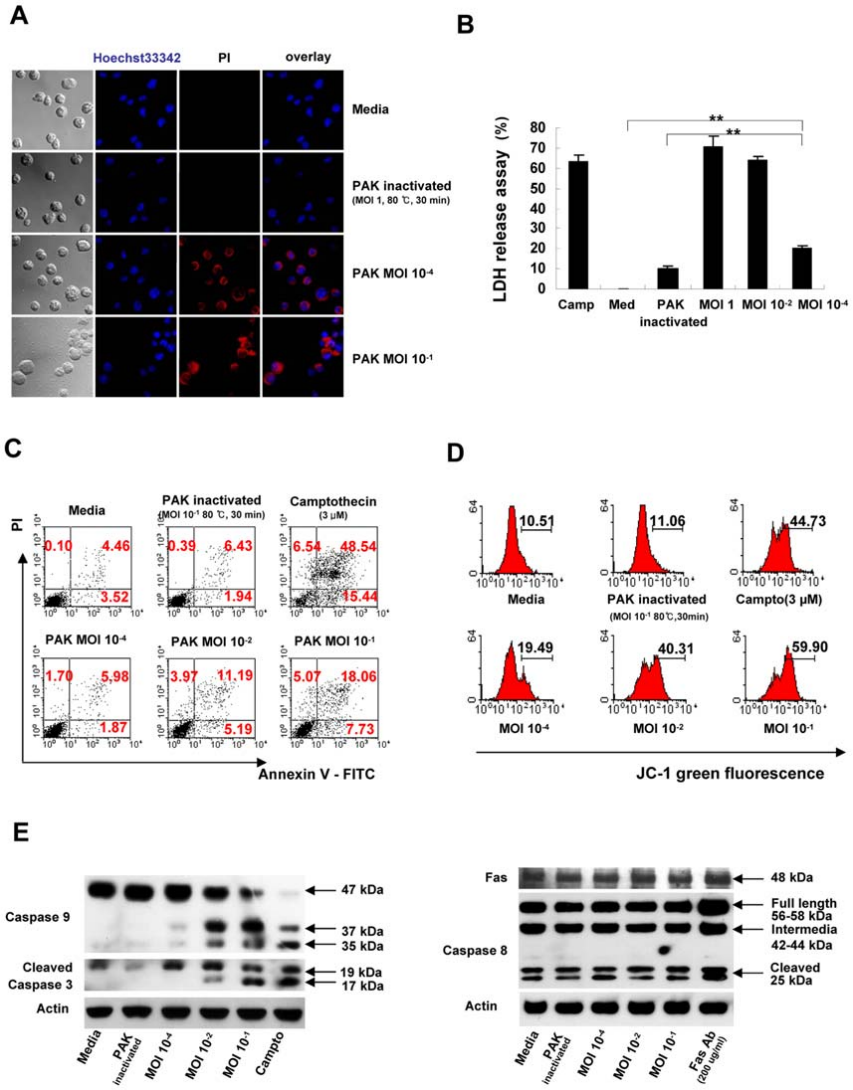
PAK induces cell death of NK cells *in vitro*. (A) NK92 cells were infected with PAK at various MOIs. At 18 h, the cells were washed with PBS and stained with Hoechst 33342 and propidium iodide (PI) for fluorescence microscopy to analyze cell death *in vitro* (original magnification, 800×). (B) NK92 cells were infected with PAK at various MOI, and the cytotoxicity of PAK against NK cells was determined using the lactate dehydrogenase (LDH) release assay. Treatment with camptothecin (3 µM) was used as a positive control (**p<0.005). (C) Flow cytometric analysis of apoptosis as assessed by Annexin-V and PI staining. These data are representative of at least three individual experiments. (D) Changes in mitochondrial membrane potential in NK92 cells following PAK infection for 24 h. The mitochondrial potential was measured by flow cytometry using the JC-1 molecular probe. The loss of mitochondrial potential is detected as a relative loss of red (FL2) fluorescence and gain of green fluorescence (FL1) compared to controls. Note that there is a significant and dose-dependent loss of mitochondrial potential following PAK infection. Cells treated with camptothecin (3 µM) served as a positive control for apoptosis. Enhanced green fluorescence of NK92 cells indicated a decrease in mitochondrial potential. (E) NK92 cells were infected with the indicated MOI of PAK and incubated for 12 h at 37°C. Following incubation, cells were lysed in sample buffer containing 2% SDS. Proteins in whole lysates (100 µg/lane) were resolved by SDS/PAGE, electroblotted onto PVDF membranes, and probed with antibodies specific for caspase-3, -8, -9, and Fas. β-actin was included as a loading control. Camptothecin and Fas antibody served as controls for apoptosis. Results are representative of three independent experiments.

To determine whether the reduction in the number of NK cells after PAK infection was due to decreased infiltration or to direct elimination, we infected NK92 cells with PAK *in vitro* and analyzed the survival of the NK cells via microscopy and flow cytometry. As seen in Fig. 3A, infection with PAK significantly increased the number of propidium iodide (PI)-positive NK cells, whereas heat-inactivated PAK did not affect the survival of NK cells. The lactate dehydrogenase (LDH) release assay also showed that PAK directly kills NK cells in a concentration-dependent manner compared with control or inactivated PAK (Fig. 3B). In addition, flow cytometric analysis revealed that PAK infection induced both early (PI^−^/Annexin-V^+^) and late (PI^+^/Annexin-V^+^) apoptosis of NK cells in a concentration-dependent manner (Fig. 3C). Taken together, these results suggest that PAK infection directly kills NK cells via apoptosis.

### PAK induces caspase-9-dependent apoptosis of NK cells

Two caspase activation pathways are centrally involved in executing apoptotic events: the receptor-initiated, caspase-8-dependent pathway and the mitochondria-initiated, caspase-9-dependent pathway [25]. Therefore, we investigated changes in mitochondrial membrane potential after PAK infection of NK92 cells by staining them with JC1 (Molecular Probes, Netherlands). PAK infection affected mitochondrial integrity in a concentration-dependent manner, whereas heat-inactivated bacteria did not affect the mitochondrial membrane potential (Fig. 3D). Accordingly, caspase-9 and caspase-3 were activated in a concentration-dependent manner by bacterial infection, whereas caspase-8 was not significantly affected (Fig. 3E). These results suggest that PAK-induced NK cell apoptosis does not occur through a receptor-mediated pathway, but rather follows mitochondria-initiated, caspase-9-dependent mechanisms.

### PAK-induced NK cell apoptosis is independent of the type III secretion (TTS) system and of secreted toxins

**Figure 4:**
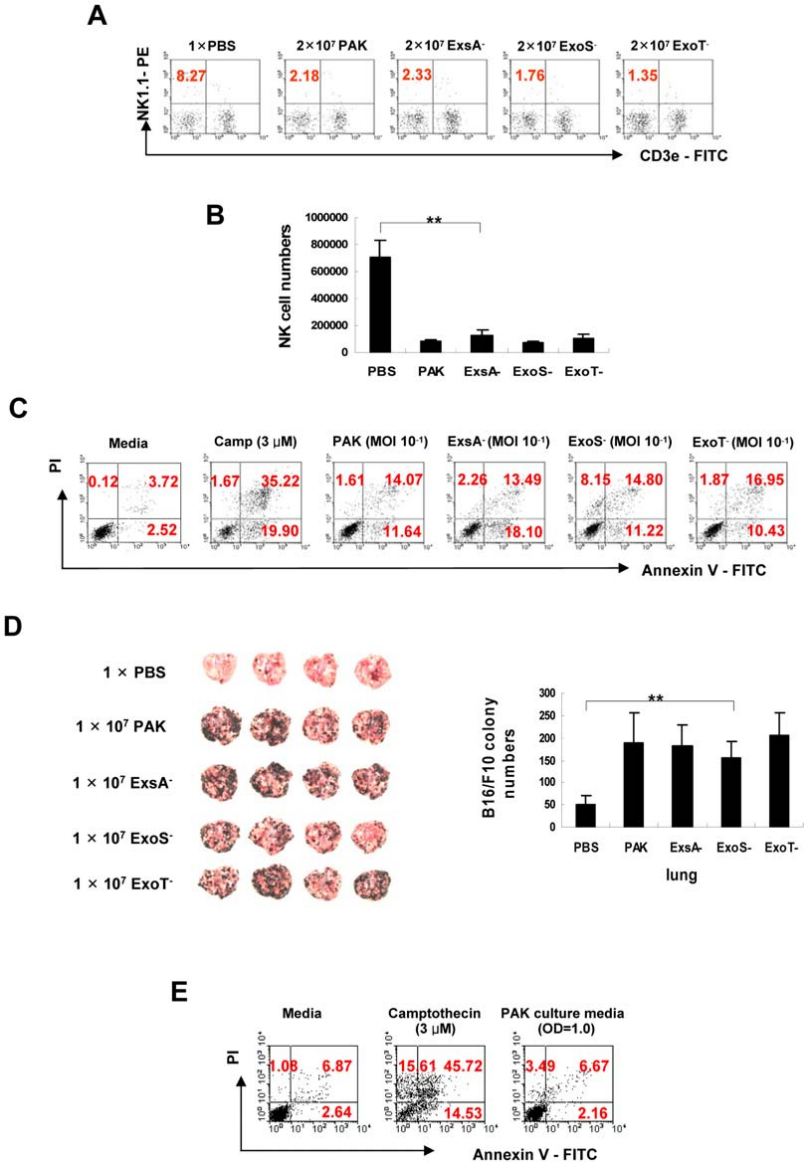
PAK*-*induced NK cell apoptosis is independent of the TTS system. Flow cytometric analysis of the population (A) and absolute number (B) of NK cells in spleens 24 h after *i.v.* injection of WT or TTS-defective mutant strains of PAK (n = 3) (**p<0.005). (C) NK92 cells were infected with WT or TTS-defective mutant strains of PAK at a MOI of 10^−1^. After 18 h, apoptosis was evaluated by flow cytometry after Annexin-V and propidium iodide (PI) staining. (D) C57BL/6 mice were inoculated *i.v.* with 1×PBS, 1×10^7^ PAK, or TTSS-defective mutants of PAK, and subsequently *i.v.-*inoculated with 1×10^5^ B16-F10 melanoma cells 2 days post-infection. Representative photographs of lungs at 2 weeks after infection (left). The numbers of surface metastatic foci were counted (right). Values presented are means±SD of triplicate determinations (n = 5) (**p<0.005). (E) WT PAK was grown in α-MEM at 37°C to an OD_600 nm_ of 1.0. The cultures were centrifuged at 6000 rpm (2613×g) for 15 minutes, and the supernatants were collected, filtered through a 0.2-micron filter, and used as a conditioned medium containing secreted bacterial toxins. NK92 cells were cultured in the conditioned medium for 24 h and analyzed for apoptosis by flow cytometry. These data are representative of at least three individual experiments.

PAK uses TTS systems to secrete several exotoxins, including Exo A, Exo T, Exo S, and Exo U, all of which are known to induce cellular apoptosis [10],[26],[27],[28]. Thus, we tested whether PAK*-*induced NK cell apoptosis was dependent on the TTS system by infecting NK cells with corresponding mutant strains of PAK. When ExoS^−^ or ExoT^−^ bacterial strain were *i.v*.-injected into C57BL/6 mice, the percentage (Fig. 4A) and absolute number (Fig. 4B) of NK cells (NK1.1^+^/CD3^−^) were prominently reduced *in vivo*, comparable to the effects of wild type (WT) strain infection. Moreover, the ExsA^−^ strain, in which the TTS system is disrupted [29], did not significantly decrease the NK cell number compared to the WT strain. This suggests that PAK-induced NK cell apoptosis was independent of the TTS system. Accordingly, induction of NK cell apoptosis by each exotoxin-deleted mutant strain was similar to that of the WT strain *in vitro* (Fig. 4C), and the mutant strains did not induce apoptosis of HeLa cells, as previously reported (Fig. S1) [26]. Furthermore, the mutant strains were still able to augment tumor metastasis in an *in vivo* animal model, similar to the WT strain (Fig. 4D). These results suggest that PAK-triggered NK cell apoptosis is TTS system-independent, unlike PAK-induced apoptosis of non-immune cells, such as epithelial and endothelial cells.

Secreted proteins from PAK, such as AP and elastase, may contribute to bacterial cytotoxicity in macrophage or NK cells [8],[23]. Therefore, we investigated the involvement of secreted toxins in NK cell apoptosis by measuring whether the bacterial culture medium was cytotoxic to NK cells. Flow cytometric analysis showed that culture medium containing secreted bacterial toxins did not affect cell viability (Fig. 4E), suggesting that PAK*-*induced NK cell apoptosis is independent of the secretion of bacterial toxins. Pyocyanin, a toxic metabolite of a certain strain of PA, has been shown to induce apoptosis in neutrophils [13],[30]. Thus, we examined whether pyocyanin is involved in NK cell apoptosis. As expected, flow cytometric analysis revealed that PA14, a pyocyanin-producing strain of PA, induced NK cell apoptosis, although the apoptosis was not as high as that produced by PAK (Fig. S2A). A pyocyanin-deficient PA strain, PA14-Phz1/2 [31] did not show any cytotoxic effect against NK cells. In addition, when NK cells were treated with pyocyanin *in vitro*, pyocyanin induced cytotoxicity against NK cells in a dose-dependent manner (Fig. S2B). These results suggest that pyocyanin may contribute to the apoptosis of PA-induced NK cell apoptosis depending on PA strains. However, the King Agar A assay [32] revealed that PAK, the strain used in this study, produced little amounts of pyocyanin (Fig. S2C), as previously reported [33]. Taken together, these results suggest that although pyocyanin seems to be able to induce NK cell apoptosis, it is not involved in the PAK-induced NK cell apoptosis observed in this study because the PAK strain may not produce sufficient pyocyanin to be toxic.

### PAK-induced NK cell apoptosis is distinct from autophagy

Autophagy is a non-apoptotic programmed cell death mechanism that involves a lysosomal pathway and has been reported to play a role in host defense against bacterial infection [34],[35]. Although the above results showed that PAK-induced NK cell death occurs via caspase-9-dependent apoptosis, we investigated the possible involvement of autophagy in PAK-induced NK cell death by examining the endogenous expression of LC3, an autophagic marker. Confocal microscopic analysis revealed that there was no change in LC3 expression in PAK-infected NK cells, while rapamycin-treated NK cells exhibited the punctuated structure of LC3, colocalized with lysosomal marker Lamp-1, which is indicative of autophagosome formation (Fig. S3). These results demonstrate that internalization of PAK did not induce autophagy in NK cells and that PAK-induced NK cell death is a distinct process from autophagy.

### Internalization of PAK by NK cells

**Figure 5:**
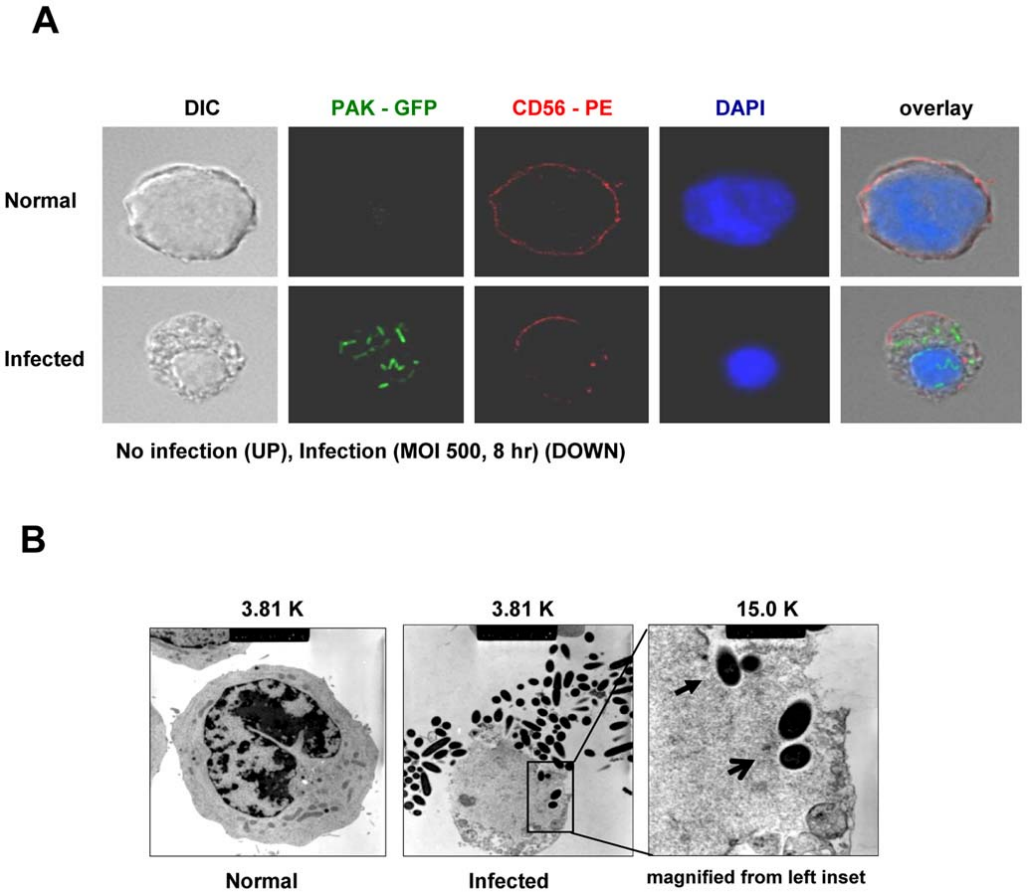
Entry of PAK into NK cells. PAK entry into NK cells was evaluated by confocal microscopy and transmission electron microscopy (TEM). NK92 cells were infected with PAK-GFP at MOI of 500 for 8 h. (A) NK cells were detected by staining with CD56-PE (membrane) and DAPI (nuclei); PAK were visualized directly by GFP fluorescence under a confocal microscope (original magnification, 600×). (B) An NK cell with internalized bacteria observed by TEM (original magnification, 3,810×). Inset: bacteria invading via a phagocytic cup formation (closed arrow) and internalized bacteria within a vacuole-like structure (open arrow) (original magnification, 15,000×).

Because the above results showed that PAK-induced NK cell apoptosis is not dependent on bacterial toxins, we hypothesized that NK cell apoptosis following PAK infection might be due to the active invasion of intact bacteria into NK cells. Thus, we constructed GFP-expressing PAK (PAK-GFP) and used confocal microscopy to investigate its localization (Fig. 5A). When NK cells were co-cultured with PAK-GFP (bottom), PAK-GFP bacteria were internalized by the NK cells (Green). Moreover, typical characteristics of apoptosis, including disruption of membrane integrity (Red) and nuclear condensation (Blue), were observed following infection. Control NK cells retained normal membrane integrity and large nuclei (top). These results suggest that PAK can cause phagocytosis-induced apoptosis of NK cells. Furthermore, transmission electron microscopy (TEM) revealed bacterial internalization and disruption of the entry site at the NK cell surface (Fig. 5B). In addition, the bacteria inside the cell were surrounded by vacuole-like structures, suggesting that PAK enters NK cells by phagocytosis. Taken together, these results show that PAK can invade NK cells and stimulate phagocytosis-induced apoptosis of non-phagocytic NK cells.

### PI3K is involved in invasion of PAK into NK cells

**Figure 6:**
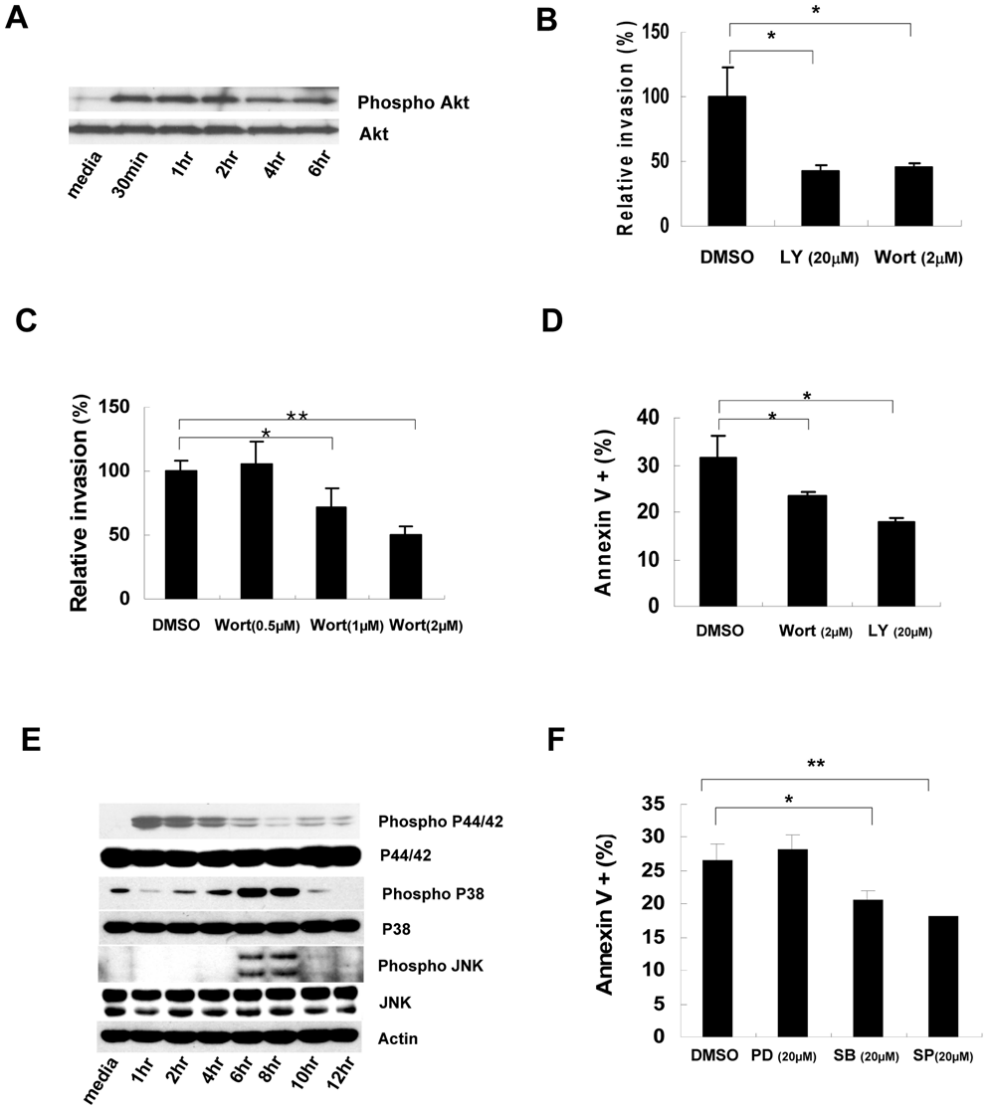
Involvement of PI3K in internalization of PAK and apoptosis of NK cells. (A) NK92 cells were infected with PAK at a MOI of 250 for various time periods. For PI3K action, p-Akt levels were monitored by immunoblot analysis. (B) NK92 cells were pretreated with PI3K inhibitors including LY294002 (LY) and Wortmannin (Wort) for 30 minutes prior to infection, and the internalization assay was performed as described in Materials and Methods. The relative invasion rate was calculated by comparing the number of internalized bacteria in the vehicle-treated control with those in inhibitor-treated samples. Assays were performed in triplicate: error bars indicate±SD (*p<0.01). (C) The invasion rate was measured using NK92 cells pretreated with various concentrations of Wortmannin (Wort) prior to PAK infection. Results are expressed as means±SD of three individual experiments (*p<0.05, **p<0.01). (D) Apoptosis in NK92 cells pretreated with the PI3K inhibitor prior to infection was assessed by flow cytometry after Annexin-V staining. Data represent means±SD of three individual experiments (*p<0.05). (E) MAP kinase activations in PAK-infected NK92 cells were monitored by immunoblot analysis using antibodies against p44/42, p38, and JNK. Results are representative of three independent experiments. (F) Involvement of MAP kinases in PAK-induced NK apoptosis was assessed by flow cytometry with Annexin-V staining of PAK-infected NK cells pretreated with various inhibitors of MAP kinases for 1 h prior to infection. Results are expressed as means±SD of three individual experiments (*p<0.05, **p<0.01). PD: PD98059 (p44/42), SB: SB203580 (p38), SP: SP600125 (JNK).

Because PI3K has been implicated in PAK internalization of non-phagocytic cells [36], we investigated the involvement of PI3K in PAK entry into NK cells. Western blot analysis demonstrated that bacterial infection activated Akt in NK cells in a time-dependent manner (Fig. 6A), suggesting the participation of PI3K in this process. Additionally, treatment with PI3K inhibitors significantly decreased bacterial entry into NK cells (Fig. 6B) and blocked internalization of the bacteria in a concentration-dependent manner (Fig. 6C). Treatment with a PI3K inhibitor also prevented PAK-induced apoptosis of NK cells (Fig. 6D), supporting the involvement of PI3K in PAK entry into NK cells.

### Invasion and cytotoxicity are sequential events in PAK-induced NK cell apoptosis

MAP kinases are critical factors for the survival or apoptosis of cells. In general, p38 and JNK are involved in cell death mechanisms, whereas Erk1/2 is critical for cell survival [37]. We therefore investigated the involvement of MAP kinases in bacterial invasion and apoptosis of NK cells. Western blot analysis showed that JNK and p38 were activated in the later stage after infection, whereas Erk1/2 was gradually deactivated (Fig. 6E). This suggests that PAK infection induced death signals while attenuating survival signals in NK cells. Interestingly, the activation of JNK and p38 occurred when Erk1/2 disappeared: p38 was gradually activated during infection, whereas JNK suddenly appeared at 6 h post-infection and PI3K was activated earlier (Fig. 6A, E). These results suggest that MAP kinases may affect the survival of NK cells, whereas PI3K might be involved in bacterial invasion. Additionally, treatment with inhibitors of p38 and JNK decreased the bacterially-induced apoptosis of NK cells, whereas the Erk1/2 inhibitor did not affect the survival of NK cells infected with PAK (Fig. 6F).

### ROS is a critical factor in PAK-induced NK cell apoptosis

**Figure 7:**
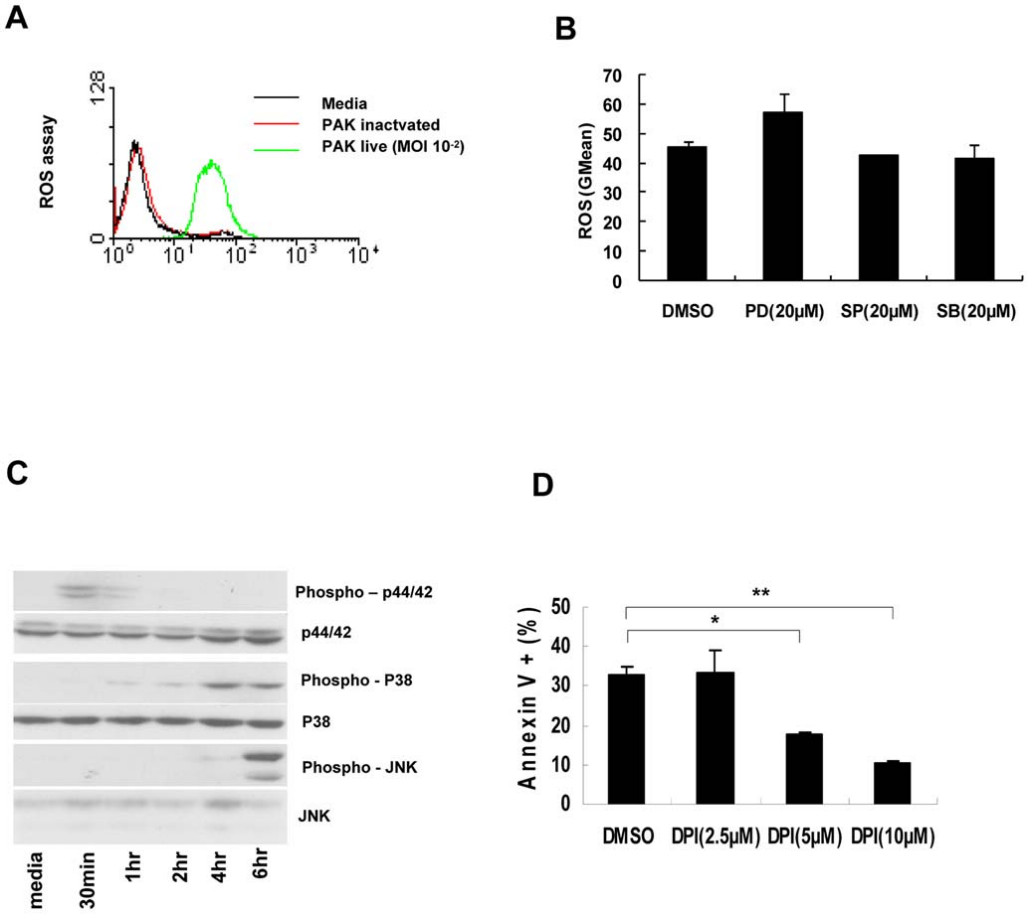
NK cell apoptosis by PAK is dependent on ROS. (A) NK92 cells were infected with PAK at MOI of 10^−2^ for 18 h. Then the cells were treated with dichlorodihydrofluorescein diacetate (DCFDA) for 30 min. Intracellular ROS production was monitored fluorometrically by oxidation of DCFDA. Data are representative histograms from three individual experiments. (B) NK92 cells were pretreated with MAP kinase inhibitors for 1 h followed by PAK infection for 16 h. Intracellular ROS production was measured by FACS analysis. (C) Effect of ROS on MAP kinase activation. NK92 cells were treated with H_2_O_2_ (50 µM) for indicated time. MAP kinase activation was monitored by immunoblot analysis using antibodies against phospho-p44/p42, p38, and JNK. (D) NK92 cells were pretreated with diphenyleneiodonium chloride (DPI) at the designated concentration for 1 h, and then infected with PAK at a MOI of 300. Apoptosis was analyzed by flow cytometry at 6 h post-infection with Annexin-V staining. Assays were performed in triplicate: error bars indicate±SD (*p<0.05, **p<0.01).

ROS has been identified as a critical factor in phagocytosis-induced apoptosis [38]. MAP kinases such as JNK are involved in ROS generation, which ultimately leads to apoptosis [12]. Therefore we measured ROS generation during PAK-induced NK cell apoptosis. A significant increase in ROS levels was observed after PAK infection of NK cells, whereas heat-inactivated PAK did not affect ROS generation (Fig. 7A). In addition, pretreatment of NK cells with inhibitors of various MAP kinases did not block the ROS generation induced by PAK invasion (Fig. 7B). However, treatment of H_2_O_2_ gradually induced MAP kinase activation in NK cells (Fig. 7C). These results suggest that ROS is an upstream signal of MAPK activation in PAK-induced NK cell apoptosis. Moreover, DPI, a NADPH oxidase inhibitor, blocked NK cell apoptosis in a concentration-dependent manner (Fig. 7D), suggesting that ROS generation is critical for NK cell apoptosis induced by phagocytosis of PAK.

## Discussion

Bacterial infection is a critical complication that may lead to prolonged hospitalization of cancer patients [6],[7],[22]. Bacteremia, the presence of viable bacteria circulating in the bloodstream, results in a reduction in the number of lymphocyte subsets [39],[40],[41], which in turn may result in tumor propagation in these patients [42]. A high percentage of patients with PAK bacteremia were observed to have cancer as an underlying disease (∼50%) [7].

Several immune cell types, including dendritic cells, macrophages, neutrophils, and certain T cells, are involved in clearing PAK infection [20],[43],[44],[45]. However, PAK has its own strategies for eluding the immune system of the host, including the elimination of immune cells [15]. For example, PAK kills principal effector immune cells, such as macrophages and neutrophils, by inducing phagocytosis-induced apoptosis [28],[46].

Although NK cells are the primary cell type responsible for innate immunity, the role of NK cells in the clearance of PAK is controversial. For example, it has been reported that NK cells may negatively regulate PAK clearance via the production of cytokines, which in turn can affect the activities of neutrophils directly involved in splenic bacterial clearance [20]. On the other hand, NKG2D-expressing cells, including NK, T, and NKT cells, have also been implicated in the clearance of PAK [21],[47]. In addition, a recent study showed that IFN-γ production by NK cells is involved in the elimination of PAK [19]. In this regard, we speculate that interactions must occur between PAK and NK cells, the results of which can be detrimental or beneficial to the host.

In the present study, we found that PAK directly invades NK cells and kills them by inducing apoptosis. The fact that adoptive transfer of NK cells attenuated metastasis after PAK infection implies that the reduction of NK cells by PAK may be a major reason for relapse in cancer patients. PAK-induced apoptosis of host cells such as macrophages, neutrophils, and epithelial cells has been studied extensively [12],[13],[14]. In general, the death of these cells occurs through the TTS system and involves ExoS, ExoT, and Exo U [26],[27],[28]. The TTS system delivers cytotoxins directly from the bacterium to the host through a contact-dependent mechanism that eventually kills the host cell. In our study, various PAK mutants that lacked TTS toxins exhibited the capacity to kill NK cells. This suggests that PAK-induced apoptosis may not utilize the TTS system. Instead, PAK may elicit phagocytosis-induced cell death (PICD) of NK cells via caspase and MAP kinase activation, as well as via ROS generation, as observed for professional phagocytes [38],[48],[49]. A number of bacteria, including Salmonella, Shigella, Mycobacterium, and PAK, induce PICD of leukocytes [50],[51], and PICD of phagocytic leukocytes has emerged as an important mechanism in the pathogenesis of bacterial infections [28],[38],[52]. Notably, the outcome of PICD may be detrimental or beneficial to the host's immune response. For example, pathogen-induced apoptosis of macrophages may adversely affect the immune response, whereas that of neutrophils may be critical for clearing infections [38],[46]. This topic requires further investigation.

NK cells are the lymphocytes that play an important role in immune surveillance against tumors [53]. Intravenous injection of B16-F10 melanoma cells is a common model for hematogenous metastases, and NK cells have been shown to reduce tumor lung metastasis in the murine cancer models. Therefore, NK cells are regarded as one of the main effector cells that are responsible for the early response against tumors [54]. The fact that the observed reduction of NK cells following PAK infection exacerbated the metastasis of cancer cells strongly suggests that decreased NK cell number may be a major reason for relapse in cancer patients. Interestingly, bacteria were rarely present in the spleen or the lung at 48 h post infection (Fig. S4), suggesting that they may be cleared by the systemic immune system. Thus, augmentation of tumor metastasis in PAK-infected mice implies that the initial induction of NK cell apoptosis by PAK may be sufficient to cause impairment of host defense against bacterial infection in cancer patients. Blood NK cells have been reported to play critical roles in preventing tumor cell localization to the lung [54].

In summary, we have shown that PAK infection induced metastasis of cancer cells in a tumor-bearing animal model and that adoptive transfer of NK cells restored the tumor clearance ability of the host. We further showed that PAK actively invaded NK cells via PI3K activation and that phagocytosis of PAK by NK cells induced mitochondria-mediated apoptosis via activation of caspase-9, JNK, p38, and ROS generation, ultimately leading to the elimination of NK cells.

## Materials and Methods

### Animals

6- to 8-week-old C57BL/6 mice were purchased from The Jackson Laboratories (Bar Harbor, ME) and maintained under specific pathogen-free conditions with standard temperature and lighting. Mice were given food and water *ad libitum*. All studies were approved by the institutional review board (KRIBB Institutional animal care and use committee/KRIBB-IACUC, approval number: KRIBB-AEC-9027), and all procedures were performed in accordance with institutional guidelines for animal care.

### Cells

All cells were purchased from the American Type Culture Collection (ATCC). NK92 (CRL-2407^TM^) cells were cultured in α-Minimum Essential Medium (MEM) (GIBCO, 12561) supplemented with 20% fetal bovine serum (FBS) (Hyclone, SH30396.03) and 10 ng/ml of IL-2 (PEPROTECH Asia, 200–02). HeLa S3 (CCL-2.2^TM^) and B16-F10 (CRL-6475^TM^) cells were cultured in Dulbecco's Modified Eagle's Medium (DMEM) (GIBCO, 11995) containing 10% FBS.

### Bacteria

PAK, PA14, and PA-phz1/2 were cultured in Bacto^TM^ Tryptic Soy Broth (TSB) (BD Biosciences, 6292241), and TTS toxin-deficient mutants of PAK, Exo A^−^ and Exo S^−^, were cultured in TSB containing 200 µg/ml of streptomycin and spectromycin. Exo T^−^ cells were cultured in TSB containing 200 µg/ml of gentamicin. After incubation for 16–18 h at 37°C, bacteria were harvested by centrifugation at 6000 rpm (2613×g) for 15 min. The bacteria were washed 3 times with phosphate buffered saline (PBS) and used to infect cells *in vivo* via *i.v.* injection or *in vitro*. Bacterial number were calculated by measuring the absorbance at 600 nm and absorbance of 0.5 AU represented 3×10^8^ cfu/ml.

### Construction of GFP-expressing PAK

A plasmid with p519ngfp (ATCC 87453) was introduced into PAK by electroporation using the Gene Pulser II (Bio-Rad, Hercules, CA, USA) set at 2.5 kV, 25 µF, and 200 Ω in 0.2-cm cuvettes, according to the manufacturer's instructions. The resulting clones were streaked onto TSB plates with kanamycin (500 µg/ml), and colonies were selected using direct fluorescence. Colonies were further amplified in kanamycin-supplemented medium. Invasion ability and cytotoxicity of PAK-GFP were verified by flow cytometry (Fig. S5).

### Flow cytometric analysis of mouse spleen lymphocytes

Splenocytes were isolated from each mouse 24 h after tail vein injection of PBS or PAK and the population of lymphocytes was measured by flow cytometry. The antibodies used in these experiments including NK1.1-PE (553165), CD3e-FITC (553062), CD11b-PE (557397), Ly-6G/Ly6C-FITC (553126), CD45R/B220-PE (553090), and CD19-FITC (553785), were purchased from BD Biosciences.

### Lactate dehydrogenase (LDH) release assay

NK92 cells were infected with PAK at various MOI (multiplicity of infection) and supernatants were harvested after 16–18 h to detect LDH release. NK92 cell death was determined by measuring lactate dehydrogenase activity with the CytoTox 96 assay kit (Promega, Madison, WI).

### Apoptosis assay

Pyocyanin (10009594) was purchased from Cayman Chemical (Michigan, USA). For the flow cytometric assay, the Annexin V-FITC Apoptosis Detection Kit I (556547) and Annexin V-PE (556422) were purchased from BD Biosciences. For western blot analyses of caspases, antibodies against caspase-8 (Calbiochem), caspase-9 (Cell Signaling, Danvers, MA), and cleaved caspase-3 (Asp175) (Cell Signaling) were employed. FAS (B-10) (Santa Cruz Biotechnology), (S)-(+)-camptothecin (C9911) and etoposide (E1383) from Sigma–Aldrich (St. Louis, MO) were used as positive controls for the induction of apoptosis.

### Assessment of mitochondrial potential

To determine mitochondrial membrane potential, infected cells were washed twice with 1×PBS and incubated with 1 µg/ml of the JC-1 stain (Molecular Probes-Invitrogen, T3168) for 30 min at 37°C. Cells were washed in PBS and subjected to flow cytometric analysis. The red fluorescence of JC-1 aggregates represents the intact mitochondrial membrane potential, whereas the green fluorescence of JC-1 monomers is indicative of mitochondrial depolarization.

### Measurement of ROS

ROS production was analyzed by labeling cells with 20 µM 2′,7′-dichlorodihydrofluorescein diacetate (DCFDA) (Invitrogen, C400) for 30 min at 37°C in the dark. Samples were examined by fluorescence-activated cell sorter (FACS) analysis, and the results were analyzed using CellQuest software (Becton Dickinson, San Jose, CA).

### Confocal fluorescence microscopy

Confocal fluorescence images were obtained using a Zeiss LSM510NLO confocal scan head mounted on a Zeiss Axiovert 200 M inverted microscope with a 63× objective. Sequential excitation at 488 nm and 543 nm were produced by argon and helium-neon gas lasers, respectively. The emission filters BP500–550 and LP560 were used to collect green and red in channels one and two, respectively. After sequential excitation, green and red fluorescence images of the same cell were acquired using Laser Sharp software. Images were analyzed using Zeiss software. For the autophagy assay, cells were fixed and washed with the BD Cytofix/Cytoperm^TM^ Fixation/Permeabilization Kit (BD Biosciences, 554714) and blocked in 2% BSA/PBS for 30 min. After incubation with anti-LC3 antibody (MBL, PM036) and anti-LAMP1 antibody (SANTA CRUZ, sc-18821) for 3 h at 4°C, cells were washed three times and incubated in fluorescein-anti-rabbit IgG (VECTOR, FI-1000), and PE-anti-Mouse IgG1 (BD Bioscience, 550083) antibodies for 1 hr at 4°C to visualize LC3 and LAMP1, respectively. Cells were washed three times counterstained with DAPI (VECTOR, H-1500), and imaged with a confocal fluorescence microscope.

### Transmission electron microscopy (TEM)

TEM was carried out to further visualize the intracellular internalization of PAK. NK92 cells were infected with PAK at a MOI of 500 for 8 h, and the cells were washed twice with cold PBS, and fixed overnight in 2.5% glutaraldehyde. On the following day, cells were rinsed three times with PBS and post-fixed in 1% osmium tetroxide (O_S_O_4_) for 1 h. The cells were dehydrated using increasing concentrations of ethanol (30%, 50%, 75%, 100%, 100%, 10 minutes each) and embedded in Polybed 812 epoxy resin (Polysciences). Sections (60 nm thick) were cut and stained with 4% aqueous uranyl acetate for 15 min, followed by lead citrate for 7 min. Samples were viewed by electron microscopy.

### Mitogen-activated protein (MAP) kinase signaling assay

For analysis of mitogen-activated protein (MAP) kinase signaling pathways, phospho-specific antibodies against ERK1/2, JNK/SAPK and p38 were used to detect expression in whole cell lysates by western blotting. All antibodies were purchased from Cell Signaling, inhibitors of MAP kinases were purchased from Calbiochem.

### PAK internalization assay

NK92 cells were infected at a MOI of 100 at 37°C for 3 h. Cells were washed 3 times with PBS and incubated with experimental medium containing gentamicin (200 µg/ml) for 1 h at 37°C to kill extracellular bacteria. The cells were washed again with PBS and lysed in 0.25% Triton X-100 for 10 min at room temperature. The released intracellular bacteria were enumerated by plating on Tryptic Soy Agar plates.

### *In vivo* metastasis assay

PBS (50 µl) or various doses of PAK were injected into C57BL/6 mice via *i.v.* injection. At 2 days post-infection (DPI), B16-F10 cells in the exponential growth phase were harvested and injected *i.v.* into the mice. For the adoptive transfer experiments, 1×10^6^ NK cells in 50 µl PBS were injected into the mice at 3 DPI. At 12 DPI, the mice were sacrificed and the number of tumor cells in the lungs were enumerated.

### Cytotoxicity assay

Splenocytes and NK92 cells were activated with IL-2 (20 ng/ml) for 12 hrs and infected with live or heat-inactivated (80°C, 30 min) PAK. After 12 h, cells were treated with gentamicin (200 µg/ml) for 1 h to remove extracellular bacteria. Following this treatment, the cytotoxicities of splenocytes and NK92 cells were assessed using ^51^Cr labeled YAC-1 and K562 cells as target cells, respectively, at various effector/target ratios. After incubation, the plates were centrifuged, and 100 µl of the resulting supernatant was removed. Radioactivity was measured using a γ scintillation counter. Cell activity was calculated as the percent cytotoxicity, according to the following equation:
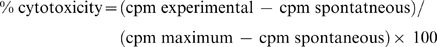



Spontaneous release of ^51^Cr was determined by incubation of target cells in the absence of effector cells: maximum release was determined by treatment with 1% Triton X-100.

### Mouse survival assay

C57BL/6 mice were *i.v.-*inoculated with 3×10^7^ PAK. Two days after infection, 1×10^7^ PFU herpes simplex virus type 1 (HSV-1) or PBS were injected *i.v.* or *i.p.* Each group was composed of 6 mice: the survival of the mice was scored 14 days after HSV-1 injection.

### Colony formation assay

Spleen and lung were aseptically removed post-infection and homogenized in 3 ml of cold PBS. Homogenates were plated on tryptic soy agar plates and incubated at 37°C for 24 h. Tissue homogenates were obtained from a minimum of 3 mice.

### Statistical analyses

All experiments were repeated at least three times. Results are presented as means±standard deviation. A student's *t* test was used to compare means and *p*<0.05 was considered as significant.

## Supporting Information

Figure S1 Effects of PAK on HeLa cell apoptosis. HeLa cells were infected with normal or TTSS-defective mutant strains of PAK at a MOI of 10^−1^. After 18 h, apoptosis was evaluated by flow cytometry using Annexin-V and propidium iodide (PI) staining.
(0.46 MB TIF)

Figure S2 Pyocyanin induces NK cell apoptosis. (A) NK92 cells were infected at various MOIs with PAK, PA14, or PA14-phZ1/2 (pyocyanin-deficient mutant of PA14) for 20 h. Camptothecin-treated cells served as a positive control. (B) NK92 cells were treated with pyocyanin for 20 h, and apoptosis was analyzed by flow cytometry. (C) 5×10^3^ CFU bacteria were cultured in 60788 King Agar A plates for 24 h, and secretion of pyocyanin was monitored.
(0.99 MB TIF)

Figure S3 Effects of PAK on NK cell autophagy. NK92 cells were infected with WT or heat-inactivated PAK (MOI 10). At 16 h, the cells were washed with PBS and subjected to fluorescence microscopy. Cells were stained with LC3 and LAMP-1 antibodies as described in Materials and Methods. Rapamycin (1 µg/ml) served as a positive control. These data are representative of at least three individual experiments.
(1.50 MB TIF)

Figure S4 Colony formation by PAK *in vivo*. Colony forming units (CFU) were determined by plating organ homogenates of infected mice. Plates were incubated for 24 h to determine the number of viable bacteria. Values represent means±SD. Data are representative of three individual experiments (n = 5).
(0.44 MB TIF)

Figure S5 Invasion of PAK into NK cells and NK cell apoptosis. NK92 cells were infected with PAK-GFP at the indicated MOI. At 12 h post-infection, apoptosis (A) and invasion (B) were analyzed with flow cytometry using Annexin V-PE and direct green fluorescence, respectively.
(0.47 MB TIF)
