# Supplementary figures and images for: Pseudomonas aeruginosa Eliminates Natural Killer Cells via Phagocytosis-Induced Apoptosis
Source: PLoS Pathog. 2009 Aug 28;5(8):e1000561. doi: 10.1371/journal.ppat.1000561 (PMC2726936; doi:10.1371/journal.ppat.1000561)

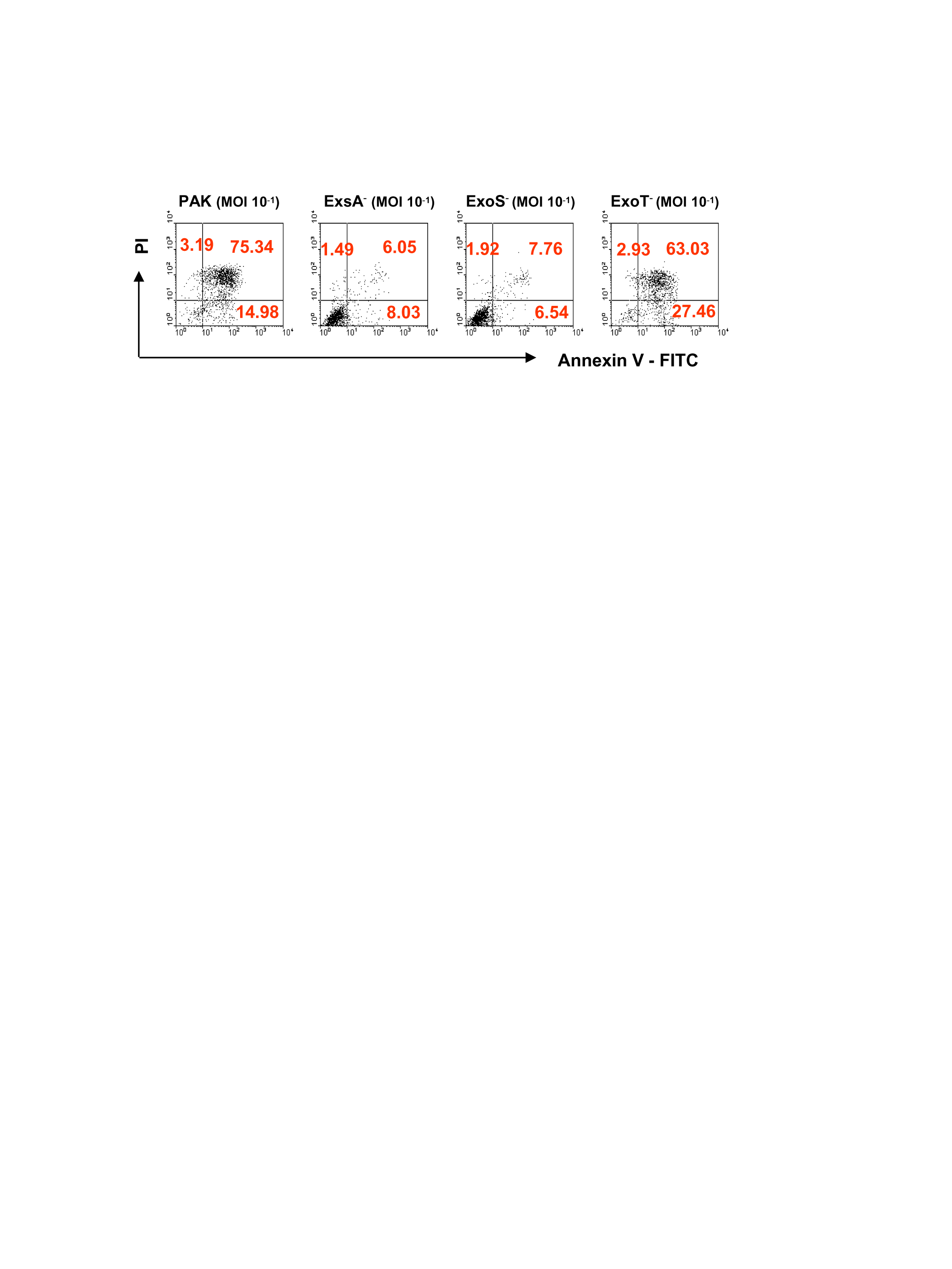

Supplement: Figure S1 — Effects of PAK on HeLa cell apoptosis. HeLa cells were infected with normal or TTSS-defective mutant strains of PAK at a MOI of 10−1. After 18 h, apoptosis was evaluated by flow cytometry using Annexin-V and propidium iodide (PI) staining. (0.46 MB TIF) [file ppat.1000561.s001.tif]

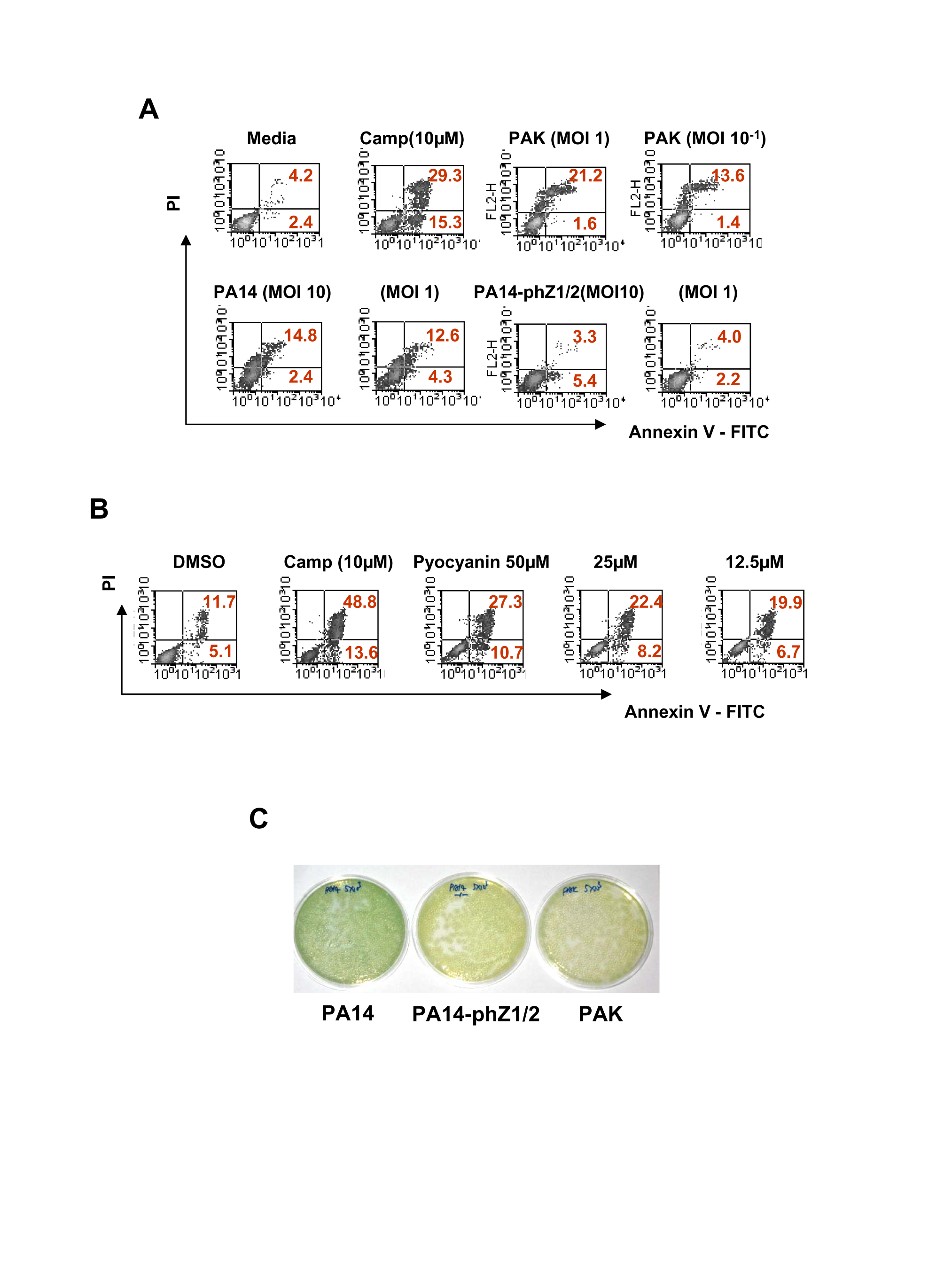

Supplement: Figure S2 — Pyocyanin induces NK cell apoptosis. (A) NK92 cells were infected at various MOIs with PAK, PA14, or PA14-phZ1/2 (pyocyanin-deficient mutant of PA14) for 20 h. Camptothecin-treated cells served as a positive control. (B) NK92 cells were treated with pyocyanin for 20 h, and apoptosis was analyzed by flow cytometry. (C) 5×10^3^ CFU bacteria were cultured in 60788 King Agar A plates for 24 h, and secretion of pyocyanin was monitored. (0.99 MB TIF) [file ppat.1000561.s002.tif]

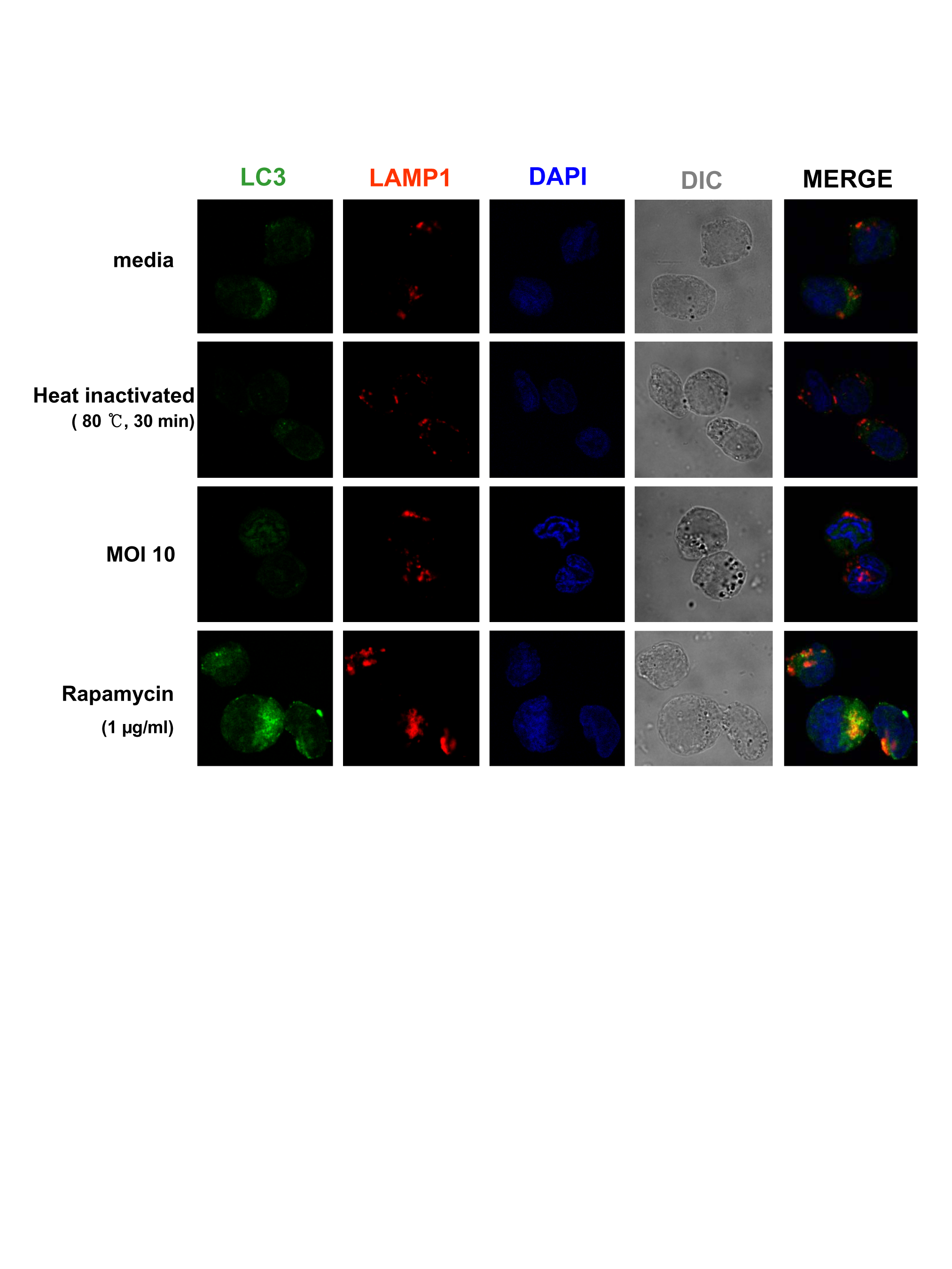

Supplement: Figure S3 — Effects of PAK on NK cell autophagy. NK92 cells were infected with WT or heat-inactivated PAK (MOI 10). At 16 h, the cells were washed with PBS and subjected to fluorescence microscopy. Cells were stained with LC3 and LAMP-1 antibodies as described in Materials and Methods. Rapamycin (1 µg/ml) served as a positive control. These data are representative of at least three individual experiments. (1.50 MB TIF) [file ppat.1000561.s003.tif]

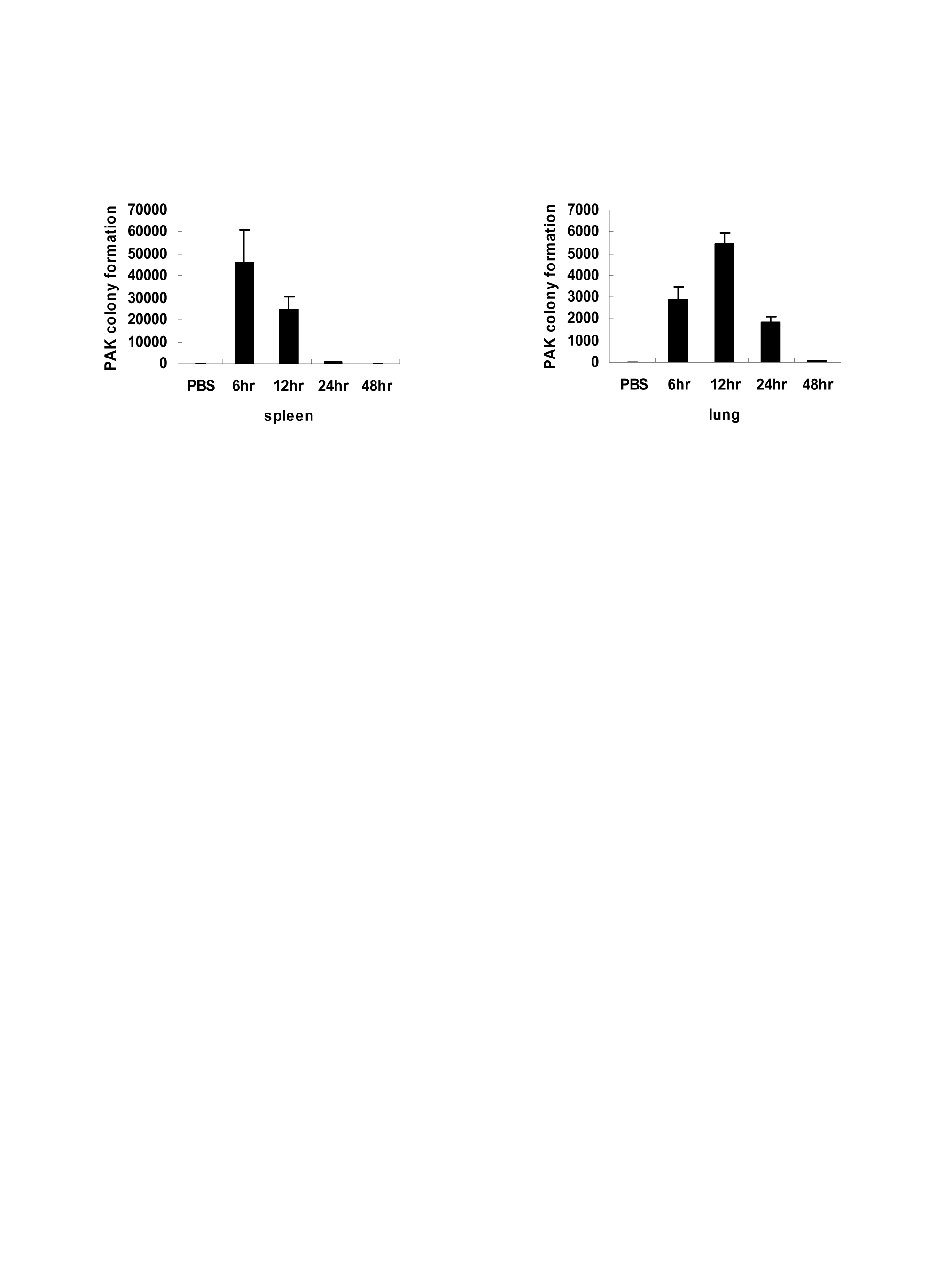

Supplement: Figure S4 — Colony formation by PAK in vivo. Colony forming units (CFU) were determined by plating organ homogenates of infected mice. Plates were incubated for 24 h to determine the number of viable bacteria. Values represent means±SD. Data are representative of three individual experiments (n = 5). (0.44 MB TIF) [file ppat.1000561.s004.tif]

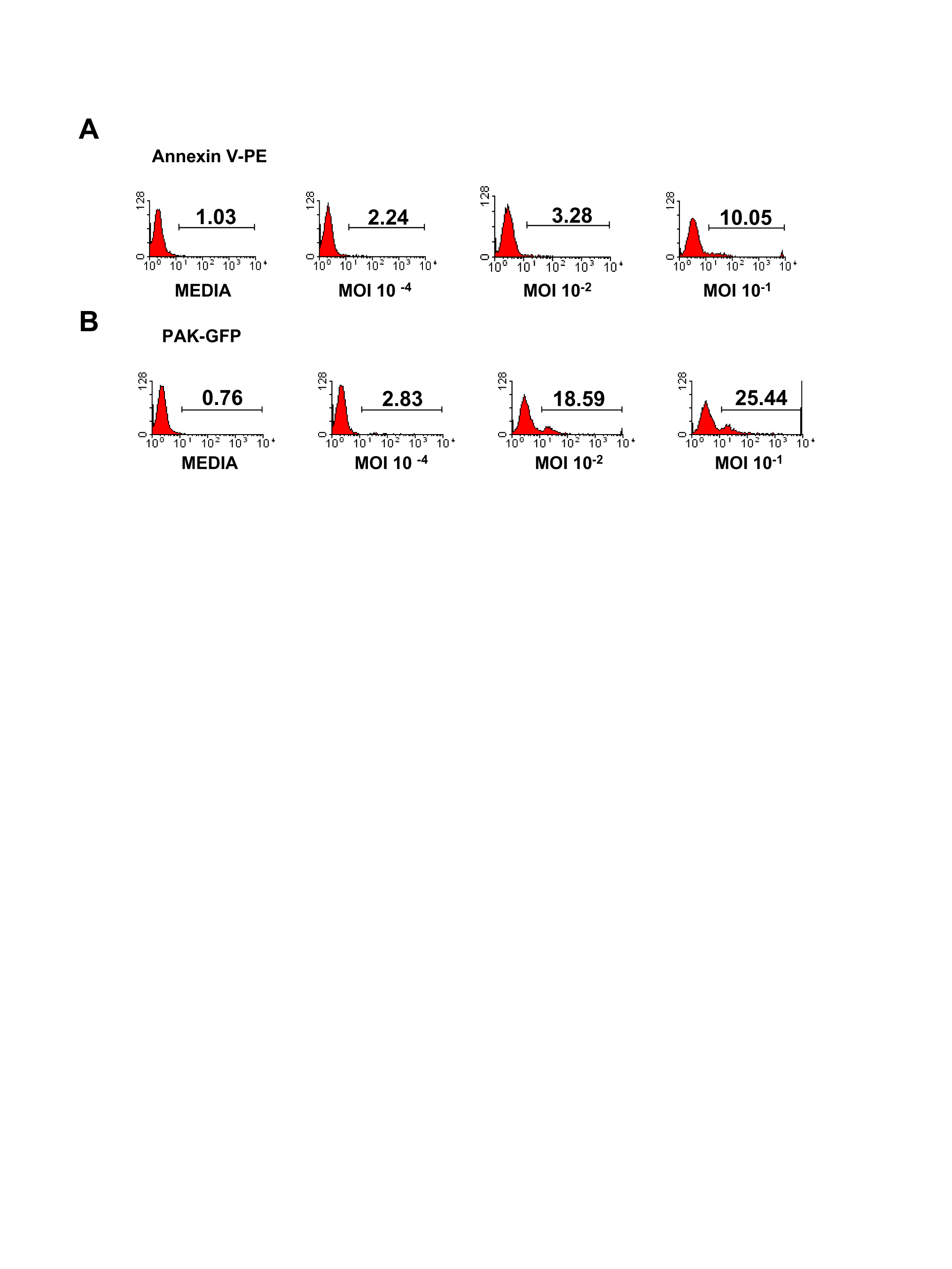

Supplement: Figure S5 — Invasion of PAK into NK cells and NK cell apoptosis. NK92 cells were infected with PAK-GFP at the indicated MOI. At 12 h post-infection, apoptosis (A) and invasion (B) were analyzed with flow cytometry using Annexin V-PE and direct green fluorescence, respectively. (0.47 MB TIF) [file ppat.1000561.s005.tif]
